# Barriers to recommendation of integrative oncology modalities in supportive care: a MASCC/SIO survey

**DOI:** 10.1007/s00520-026-11020-2

**Published:** 2026-07-22

**Authors:** Alexandre Chan, Dalia Kagramanov, Reem Nasr, Chioma Asuzu, Ting Bao, Yin Ting Cheung, Jung Hye Kwon, Judith Lacey, Richard T. Lee, Maryam Lustberg, Beatrice M. Ohaeri, Santosh Rao, Enrique Soto-Perez-de-Celis, Claudia M. Witt, Ana Maria Lopez

**Affiliations:** 1 Department of Clinical Pharmacy Practice, University of California Irvine, 802 W Peltason Drive, Irvine, CA 92697-4625 USA; 2 Department of Guidance and Counselling, University of Ibadan, Ibadan, Nigeria; 3 Zakim Center for Integrative Therapies and Healthy Living, Dana-Farber Cancer Institute, Boston, MA USA; 4 School of Pharmacy, Faculty of Medicine, The Chinese University of Hong Kong, Hong Kong SAR, China; 5 Division of Hemato-Oncology, Department of Internal Medicine, Chung-Ang University, Gwangmyeong Hospital, Chung-Ang University College of Medicine, Seoul, South Korea; 6 Department of Supportive Care and Integrative Oncology, Chris O’Brien Lifehouse Comprehensive Cancer Centre, Camperdown, NSW Australia; 7 Cherng Family Center for Integrative Oncology, City of Hope Comprehensive Cancer Center, Duarte, CA USA; 8 Breast Cancer Center, Smilow Cancer Hospital, Yale University, New Haven, CT USA; 9 College of Medicine, University of Ibadan, Ibadan, Nigeria; 10 University Hospitals Seidman Cancer Center, Cleveland, OH USA; 11 Department of Geriatrics, Instituto Nacional de Ciencias Médicas y Nutrición Salvador Zubiran, Mexico City, Mexico; 12 UC Health Diane O’Connor Thompson Breast Center, Auschutz Medical Campus, University of Colorado, Aurora, IL USA; 13 Institute for Complementary and Integrative Medicine, University Hospital Zurich, Zurich, Switzerland; 14 Sidney Kimmel Comprehensive Cancer Center, Thomas Jefferson University, Philadelphia, PA USA

**Keywords:** Integrative oncology, Cancer supportive care, Oncology, Cancer, International perspectives

## Abstract

**Purpose:**

Integrative oncology (IO) modalities aim to improve the quality of life of persons with cancer, including strategies to manage treatment and disease-related symptoms. As evidence increases to include IO in supportive care, interest grows globally. Understanding barriers to IO recommendation is key to facilitating its uptake.

**Methods:**

Members of the Multinational Association of Supportive Care in Cancer (MASCC) and the Society for Integrative Oncology (SIO) were invited to complete an online survey in Fall 2023 assessing barriers to IO recommendation on a 5-point scale. Descriptive statistics assessed demographic data and general counts of scaled agreement, while logistic regression analysis evaluated perceived barriers in relation to relevant covariates.

**Results:**

Over 80% of respondents believed that healthcare professionals’ (HCP) lack of knowledge with IO and lack of insurance/out-of-pocket costs were barriers to recommending IO. Approximately 80% agreed that lack of referral pathways was a logistical barrier and 85% reported lack of confidence in IO as an individual-level barrier. Respondents from Europe were 2.2 times more likely to agree with the perceived barrier of HCP knowledge, compared to respondents from North America (OR 2.22, 95% CI 1.05–4.67, *p* = 0.036). Lastly, compared to general physicians, integrative professionals were more likely to report perceptions that IO would delay conventional care delivery (OR 3.62, 95% CI 1.56–8.43, *p* = 0.0003).

**Conclusions:**

Results of this study highlight important barriers and facilitators of IO integration into standard supportive care globally. These findings represent perspectives from an international sample of MASCC and SIO members and may not apply to all oncology professionals. Further research is necessary to understand the global roadblocks that exist among practitioners interested in IO use.

**Supplementary Information:**

The online version contains supplementary material available at 10.1007/s00520-026-11020-2.

## Introduction

Integrative oncology (IO) is a growing system of care that aims to address the full scope of a patient’s physical, emotional, and spiritual needs by bridging together evidence-based complimentary therapies with standard cancer treatments [1–3]. This holistic model seeks to enhance clinical outcomes and improve overall quality of life for individuals affected by cancer, supporting individuals in their cancer journey before, during, and after treatment, with a focus on patient empowerment [3].

Common modalities within IO include lifestyle modifications, mind-body therapies such as mindfulness-based interventions, acupuncture, yoga, and aromatherapy [4–7]. As interest in IO has grown globally, evidence continues to expand on the efficacy of these modalities for cancer-supportive care, furthering interest in the use of IO among patients and healthcare professionals (HCP) [8, 9].

A study conducted in 2019 found that 60–80% of oncologists have recommended at least one IO modality to patients (most commonly exercise, nutrition, and psychosocial support), while other data from healthcare professionals specializing in IO modalities report even higher use (above 80%) and referral to complimentary therapies in their cancer care practices [10]. Although support for including IO in cancer is trending upward, its utilization among patients and practitioners continues to be limited by several barriers. Key obstacles identified include limited provider training and knowledge, cost and insurance coverage, lack of communication between provider and patient, and regulatory constraints [11]. Geographical factors, such as healthcare system regulations, educational practices for practitioners, and the cost of care in different regions, can further influence these barriers by limiting access. To address these challenges and support the broader integration of IO, it is crucial to identify and understand the key factors that hinder its uptake.

Therefore, the primary objective of this study was to evaluate the opinions of international members of the Multinational Association of Supportive Care in Cancer (MASCC) and the Society for Integrative Oncology (SIO) regarding the perceived barriers to the use of IO in cancer supportive care. By gathering these insights, this study aims to inform future research and initiatives that will promote the broader adoption of IO.

## Methods

### Survey design

A cross-sectional survey was distributed to active members of the Multinational Association for Supportive Care in Cancer (MASCC) and the Society for Integrative Oncology (SIO). MASCC is an international organization of healthcare providers and researchers focused on the study of supportive care in cancer; SIO is a multidisciplinary professional society dedicated to advancing the use of evidence-based integrative medicine in cancer treatment. A total of 13 MASCC and SIO members reviewed the survey questions prior to an appropriateness assessment by the MASCC Executive Committee. Two representatives from the African Organization in Research and Training in Cancer (AORTIC) participated in the review. The survey was then circulated to task force members, as well as the MASCC board of directors, for content validity before the survey was finalized. This study was approved by the Institutional Review Board at the University of California, Irvine (Protocol #3392), and the IRB granted a waiver of informed consent.

#### Inclusion/exclusion criteria

Any active MASCC and SIO members in 2023 were eligible for participation and received an invitation to complete the survey via email. There was a total of 2129 MASCC members across 70 countries and 258 SIO members at the time the survey was distributed. Members of MASCC include physicians, nurses, dental professionals, pharmacists, dieticians, physiotherapist, psychologists, and other healthcare professionals—all whom undergo mandatory verification prior to joining the organization. SIO is a multidisciplinary community of oncologists, palliative and supportive care physicians and other physicians, nurses, psychologists, social workers, nutritionists, complementary therapy practitioners, naturopathic doctors, herbalists, acupuncturists, yoga therapists, massage therapists, among other HCP and researchers. SIO members include those in academic and healthcare institutions, small businesses, and corporations, as well as individual practitioners.

### Data collection and survey information

Invitations to participate in the voluntary web-based survey were disseminated via an initial email containing a survey link sent to MASCC and SIO members in June 2023, followed by two reminder emails, with the survey remaining open from June to August 2023. No incentives were provided for participation, and the survey platform was open and not password protected. Of 2714 invited members, 431 responded to the survey (response rate 15.9%), of whom 282 completed the barriers section (completion rate among respondents 65.4%). Completion of the survey implied consent to participate. View and participation rates could not be calculated because unique survey page visitation data were not available.

The 22-question survey was developed through an iterative process by an interprofessional panel of MASCC and/or SIO members (Supplementary File 1). The survey was built in Qualtrics (Irvine, CA), a secure online data capture platform. It was pilot tested and finalized. The survey took participants approximately 15 to 20 min to complete. Items were not randomized or alternated, adaptive questioning was not utilized, and completeness checks and review of answers were not included in the survey. The information collected in this survey that is pertinent to this study were as follows: participant demographic characteristics, perceived barriers of health professionals, and patients in relation to IO use for supportive care. The results presented in this study specifically explore the participant demographic characteristics and perceived barriers section of the survey.

The first section included nine items inquiring about the demographic characteristics of the participant (i.e., age, country of practice, professional role(s), and time worked in professional role(s)). Questions were asked in a “fill-in-the-blank” or “select which applies” manner.

The final section assessed agreement with several listed barriers regarding health professional’s recommendations of IO for supportive care in a practice setting, as well as listed barriers regarding HCP’s perceived patient concerns with IO use for supportive care in one’s practice setting. Participants were asked to rate their agreement on a 5-point scale ranging from “strongly disagree” to “strongly agree.” The types of barriers listed in the survey included clinician related barriers, integrative modality barriers, health-system barriers, treatment related barriers, and logistical barriers [1].

### Categorization of perceived barriers

A total of 27 healthcare professional perceived barriers were categorized into four domains for analysis: (1) perceived knowledge-related barriers (5 barriers), (2) perceived financial barriers (3 barriers), (3) perceived logistical barriers (9 barriers), and (4) perceived individual-level barriers (10 barriers). These categories reflect respondents’ perceptions of barriers influencing the recommendation and use of integrative oncology modalities in practice settings. Perceived knowledge-related barriers included barriers related to healthcare professionals’ knowledge, as well as respondents’ perceptions of patient knowledge or awareness of integrative oncology modalities. Perceived financial barriers encompassed perceived financial constraints affecting access or use for either providers or patients. Perceived logistical barriers included perceived health system–related limitations, such as scheduling, timing, and access. Finally, perceived individual-level barriers included respondents’ perceptions of personal-level factors that may influence uptake or recommendation, such as worry, confidence, and interest or lack of interest.

### Statistical analysis

All data were analyzed using SAS Version 9.4. Descriptive statistics were used to assess initial demographic data and general counts of scaled agreement with perceived barriers, while logistic regression analysis was used to evaluate each perceived barrier in relation to demographic and clinical characteristics. Demographic and clinical characteristics were consistent across all logistic regression models. Categorical data are presented as counts and percentages, while continuous data on demographic variables are presented as means and standard deviations.

## Results

### Demographic characteristics

Overall, 2714 recruitment emails were sent across both organizations (MASCC and SIO), with 431 responses collected (response rate of 15.9%). Of the responses, 344 (79.8%) were completed beyond the initial demographic information section and were included in the analysis. Specifically, a total of 282 respondents completed the section on perceived barriers. Table 1 summarizes the demographic characteristics of our sample. These findings reflect perspectives among surveyed MASCC and SIO members and should be interpreted within this professional context.

**Table 1:** Demographic characteristics (*N* = 344)

	Number	Percent	Mean	SD
**Age**			50.7	12.8
**Gender**				
Female	218	63.4		
Male	120	34.9		
Non-binary/other	6	1.7		
**Member of**				
MASCC	236	68.6		
SIO	50	14.5		
Both	29	8.4		
None	29	8.4		
**Years worked**			17.16	11.02
**Work sector**				
Academic	123	35.8		
Mixed public/private	39	11.3		
Public	129	37.5		
Private	53	15.4		
Region* **(work/practice in)**				
Central Asia	4	1.2		
East Asia and Pacific	106	30.8		
Europe	68	19.8		
Latin America and Caribbean	12	3.5		
Middle East and North Africa	10	2.9		
North America	118	34.3		
South Asia	18	5.2		
Sub-Saharan Africa	5	1.5		
Unspecified	3	0.9		
Country’s income level*				
High	286	83.1		
Middle-upper	29	8.4		
Lower-middle	25	7.3		
Low	1	0.3		
Unspecified	3	0.9		
**Primary profession**				
Physician (working/certified in integrative health)	85	24.7		
Physician (not working/certified in integrative health)	73	21.2		
Clinical science researcher	47	13.7		
Nurse	27	7.8		
Dentist/oral surgeon	22	6.4		
Advance practice provider	14	4.1		
Patient advocate	12	3.5		
Pharmacist	11	3.2		
Acupuncturist	9	2.6		
Physiotherapist	6	1.7		
Administrative position	5	1.5		
Other**	33	9.6		
**Secondary profession**				
Not applicable/no secondary profession	97	28.2		
Clinical science researcher	61	17.7		
Physician (working/certified in IH)	35	10.2		
Nurse	27	7.8		
Physician (not working/certified in IH)	20	5.8		
Patient advocate	12	3.5		
Advance practice provider	9	2.6		
Psycho-oncologist	8	2.3		
Trainee/student	8	2.3		
Basic science researcher	7	2.0		
Pharmacist	7	2.0		
Acupuncturist	6	1.7		
Health/lifestyle coach/counselor	5	1.5		
Traditional oriental medicine practitioner	5	1.5		
Other**	37	10.8		

*Countries’ region and income level classed according to the World Bank (12)

**Other (*n* ≤ 4 per category; see Supplementary Table A)

The majority of respondents were female (63.4%), and members of MASCC (68.6%), SIO (14.5%), or both (8.4%). The mean (SD) age was 50.7 (12.8) years and the mean (SD) number of years worked was 17.2 (11.0), with most participants working in the public (37.5%) and academic (35.8%) sectors. Overall, most respondents worked in high (83.1%) or middle-upper (8.4%) income countries and reported residence in North America (34.3%), East Asia and the Pacific (30.8%), or Europe (19.8%). The most commonly reported primary professions were physicians (44.6%) and clinical science researchers (11.9%).

### Perceived knowledge barriers

**Fig. 1:**
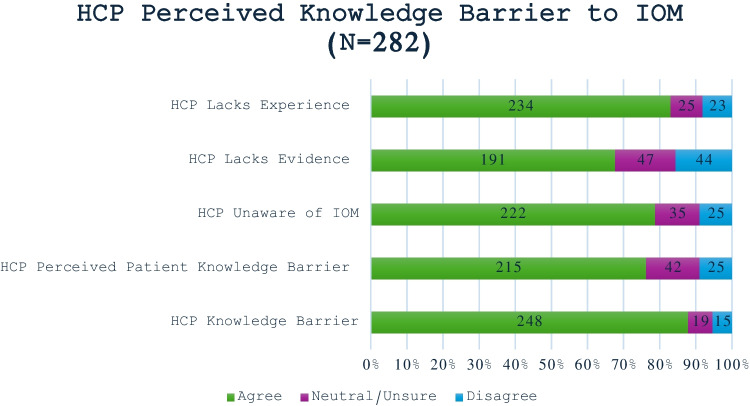
Healthcare professional agreement with perceived knowledge barriers to IOM

Among the five listed barriers related to knowledge as a perceived barrier to IO use in one’s practice setting, all participants had a majority level of agreement (Fig. 1). Over 80% of participants agreed that providers’ lack of experience and knowledge are barriers to using IO in their practice settings. Additionally, over 75% of participants also agreed that providers being unaware of IOM and their perception of a lack of knowledge from the patient were barriers to IO supportive care use in one’s practice. Lastly, over 65% agreed that a lack of evidence for the use of IO is also a barrier.

Upon further analysis exploring potential covariates to these knowledge-related barriers, results showed that age and geographical region of respondents were significant predictors of one perceiving provide knowledge to be a barrier to IO use in supportive care in one’s practice. Specifically, those older in age at the time of survey, were 2.8% less likely to have increased categorical agreement with this perceived barrier (OR 0.97 (95% CI 0.94–1.00), *p* = 0.05). Respondents from Europe were 2.2 times more likely to have increased categorical agreement with the perceived barrier of HCP knowledge to IO use, compared to respondents from North America (OR 2.22 (95% CI 1.05–4.67), *p* = 0.036).

### Perceived financial barriers

**Fig. 2:**
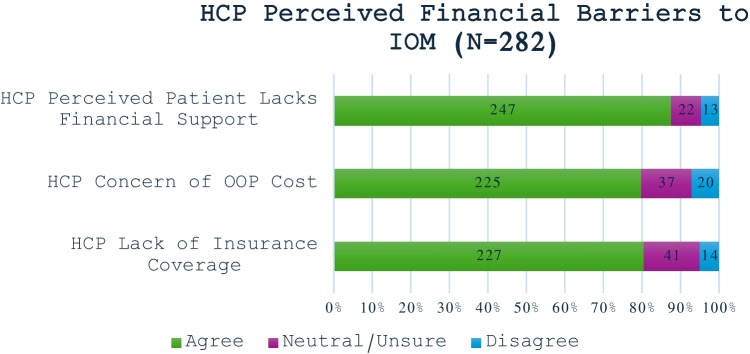
Healthcare professional agreement with perceived financial barriers to IOM use

Similar to knowledge barriers, 80% or more of participants responded that they agreed with the financial barriers listed in the survey. Providers perceived that lack of insurance coverage (82%), concerns of out-of-pocket costs (80%), and HCP perceiving patients having lack of financial support (88%) were barriers to using IO as supportive care in their practice setting. These results are displayed in Fig. 2.

The logistic regression analysis of potential predictors to these perceived financial barriers revealed no statistically significant results of demographic or clinical variables.

### Perceived logistical barriers

**Fig. 3:**
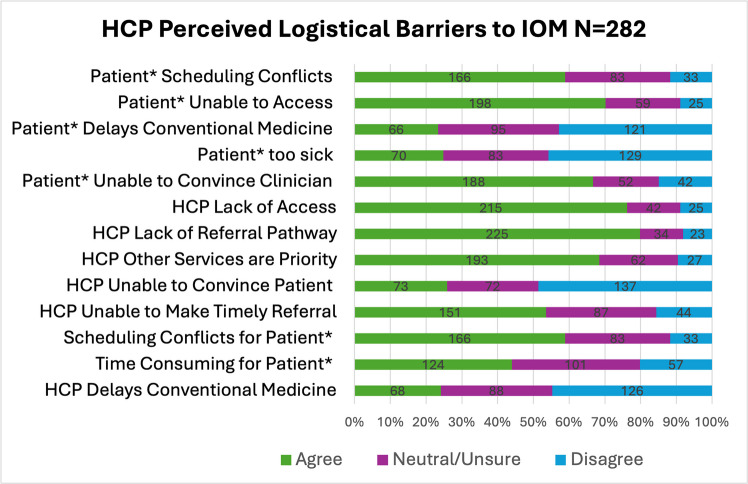
Healthcare professional perceived agreement with logistical barriers to IO use within their practice. An asterisk denotes HCP perception of patient

Eight of the 13 perceived barriers in this category had over 50% of respondents in agreement with the barrier, and 4 had over 40% that disagreed with the listed barrier to IO use. A full graph depicting each of the barriers and the percentage/counts that agreed with the barrier is shown in Fig. 3.

Among those that responded about HCP believing IO use delayed conventional medicine, results showed that one’s professional category and the number of years worked in their profession were statistically significant predictors. Those within the integrative profession were over three times more likely to agree that providers believe IO would delay conventional medicine compared to general physicians (OR 3.62 (95% CI 1.55–8.42), *p* = 0.0003). Notably, this item assessed perceived barriers to health professionals’ recommendation of integrative oncology within respondents’ practice settings, rather than respondents’ personal beliefs (Survey item 23, Supplementary File 1). Additionally, participants who have worked in their profession for 11–20 years were 2.7 times more likely to have higher categorical agreement with this perceived barrier among HCP (OR = 2.67 (95% CI 1.08–6.59), *p* = 0.03).

### Perceived individual-level barriers

**Fig. 4:**
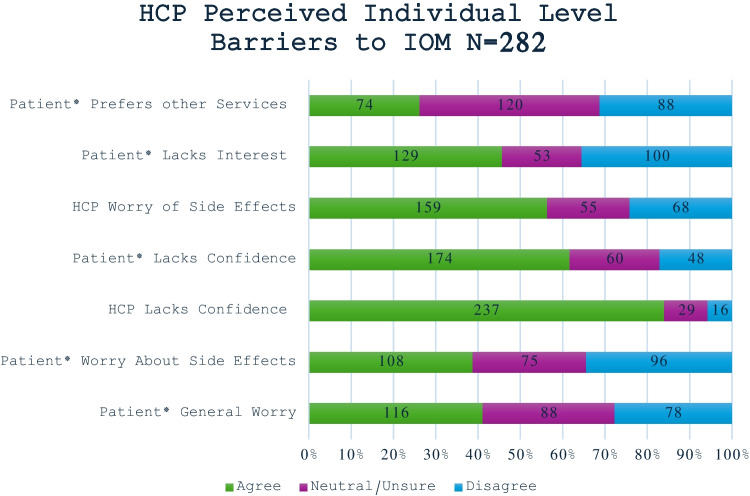
Healthcare professional perceived agreement with individual-level barriers to IO use within their practice. An asterisk denotes HCP perception of patient

Seven perceived barriers were categorized as individual-level barriers to using IO in supportive care—all depicted in Fig. 4. Importantly, these findings represent HCP respondents’ perceptions of barriers encountered in practice settings, including perceived patient-related barriers, rather than direct reports from patients themselves. Perception of providers worry about side effects, perceived lack of confidence from the patient, and perceived lack of confidence from the provider all showed over 50% agreement among participants in the study. Specifically, over 80% agreed that providers lack of confidence is a barrier to IO use. Additionally, perceived patient worries about side effects and the perception that the patient prefers other services showed less than 40% agreement.

With regard to relevant covariates to these individual-level barriers, results of the logistic regression analysis showed that HCP perceived patient general worry was predicted by age and number of years worked, while HCP perceived patient worry about side effects was predicted by the provider’s country income, region, and the underlying belief that IO is underutilized. Individuals older in age were 4% more likely to perceive patient general worry (OR 1.04 (95% CI 1.01–1.06), *p* = 0.0008). Those who have worked in their profession for 20–29 years relative to those who have worked 30 + years were 3 times more likely to perceive a barrier about general patient worry for pursuing treatment (OR 3.23 (95% CI 1.32–7.88), *p* = 0.01). A full table of all regression results is summarized in Supplementary Table 1.

## Discussion

Understanding the barriers and perceptions of integrative oncology modalities among international stakeholders is a critical step in supporting the utilization of these therapies within conventional cancer care. While interest in IO continues to rise, HCP utilization and institutional support for these modalities remain variable. Our findings highlight strong levels of agreement to knowledge-related, financial, logistical, and individual-level barriers to utilizing IO. Moreover, our results also showed that age, geographical region, years worked, and type of profession are cofactors that play a role in reported perceived barriers.

Our results of high agreement (greater than 80%) with knowledge-based barriers mirror a similar theme in the literature on IO utilization. Current data of knowledge-based barriers report limited awareness, insufficient understanding of therapeutic benefits, and a general lack of familiarity with IO modalities among both patients and HCP [11, 12]. Communication gaps between providers and patients further compound these challenges. Despite evidence suggesting that provider-led discussions are a key pathway through which patients become informed about IO options, previous studies indicate that such conversations occur infrequently. For example, Mascaro et al. reported that only 57% of patients recalled their provider discussing IO, while another study found this figure to be as low as 15% [12]. These findings underscore a critical need for improved provider education and patient-provider communication to facilitate informed decision-making around integrative approaches in care. To address knowledge-based barriers, a multi-stakeholder approach is essential. Healthcare institutions may implement IO education modules in continuing medical education programs with annual certification requirements, establish IO mentorship programs pairing experienced practitioners with newcomers, and create standardized IO resource libraries accessible through electronic health records. Professional organizations may contribute by developing competency-based IO curricula for medical and nursing schools, certification programs for HCP in evidence-based IO practices, and establishing guidelines for IO education hours in specialty training programs. At the provider level, clinicians may participate in structured IO communication training to enhance patient discussions, utilize decision-making tools and patient education materials to facilitate informed conversations, and attend interdisciplinary IO meetings to share knowledge and best practices. This comprehensive framework would address the fundamental knowledge gaps that currently impede IO integration across many levels of healthcare delivery.

Financial constraints have also been consistently identified as significant barriers to the implementation of integrative oncology (IO) services. Providers frequently cite limited institutional funding and budgetary restrictions as key challenges to incorporating IO modalities into routine care [13–15]. These findings are reiterated in our study, which surveyed international members of MASCC and/or SIO. The relatively novel stage of IO research, with a limited but growing evidence base, hinders the ability to secure reimbursement from insurance providers. As a result, patients who wish to access IO services are often faced with substantial out-of-pocket expenses, which may significantly affect equitable access to care [14, 15]. Addressing financial barriers requires coordinated efforts from healthcare systems and policymakers. Healthcare systems may develop business models demonstrating IO cost-effectiveness through reduced symptom burden and improved quality of life and pilot insurance reimbursement programs for evidence-based IO services. Additionally, policymakers may advocate for insurance coverage of IO modalities with established evidence bases, support research funding specifically aimed at IO health economics studies, and develop sliding-scale payment programs for underserved populations. Such financial strategies would help reduce the economic burden that currently limits equitable access to IO services.

Logistical barriers to IO implementation span multiple levels of the healthcare system, encompassing patient, provider, organizational, and systemic factors. Our study explored 13 related barriers, with 50% of respondents in agreement with 8 of these barriers. At the patient level, challenges include reluctance to explore unfamiliar therapies, concerns about delaying conventional treatments, limited time, and an absence of a trusting relationship with healthcare providers [11, 13]. Providers likely face constraints such as insufficient time for training, high workload demands, limited access to IO resources, ambiguous professional roles, and a lack of interdisciplinary coordination. Organizational barriers often stem from the absence of structured communication pathways between primary and secondary care, unclear allocation of provider responsibilities, and a failure to distribute resources for continuing education in IO practices. At the broader healthcare system level, limitations in standardized policies, guidelines, and supportive infrastructure further impact the integration of IO into routine care [11]. Addressing these multifaceted logistical challenges will be critical to advancing the uptake and equitable delivery of IO services. For instance, clearly defining provider roles and responsibilities within integrative oncology teams can also reduce confusion and enhance interdisciplinary collaboration. Additionally, dedicating institutional resources to support ongoing training and professional development in IO would help support clinicians with the knowledge and skills needed to confidently deliver integrative care. Overcoming logistical barriers requires systematic changes at both organizational and provider levels. Healthcare organizations may establish dedicated IO coordinators to streamline referrals, create standardized protocols for IO integration into existing care pathways, and implement electronic health record systems that facilitate IO provider communication. At the provider level, clinicians may develop clear role delineation matrices for multidisciplinary IO teams, establish regular case conferences to coordinate IO care with conventional treatments, and create patient tracking systems to monitor IO service utilization and outcomes. These structural improvements would enhance the coordination and delivery of integrated care services.

Individual-level barriers in the literature similarly to our study have focused on the lack of communication between the provider and the patient, the patient’s unwillingness to experiment with new therapies, and worry about the side effects [11, 16]. Importantly, in our study, these reflect healthcare professionals’ perceptions of patient- and provider-level barriers within practice settings, which may influence referral and implementation behaviors, rather than direct patient-reported barriers. However, relative to the other categories of barriers, limited literature has been published on individual-level factors. Further research is needed to provide a clearer understanding of what poses these barriers and how to develop an organized plan for IO implementation. Individual-level barriers may be addressed through enhanced patient education and improved provider-patient communication strategies. Patient education initiatives may include developing culturally appropriate IO educational materials addressing common concerns and misconceptions, creating peer support networks connecting patients with those with IO experience, and implementing shared decision-making tools that address individual values. Provider-patient communication may be strengthened by training providers in motivational interviewing techniques specific to IO discussions, developing conversation guides addressing common patient fears about treatment delays, and establishing protocols for regular follow-up to address emerging concerns about IO. These targeted recommendations provide concrete steps that different stakeholders may implement to address multifaceted barriers identified in our study, moving towards practical solutions for IO integration.

A consistent pattern of multifaceted barriers impeding the implementation of IO is seen in both our agreement data and in previously presented research. Both emphasize knowledge gaps as a dominant challenge, with substantial agreement among participants that providers’ lack of experience and awareness, coupled with insufficient provider/patient knowledge, hinder IO integration. Financial barriers, such as lack of insurance coverage and high out-of-pocket costs [8], were similarly cited and shown in our analyses, underscoring their widespread impact. The logistical and individual-level barriers further align, revealing shared concerns about delays in conventional treatments, lack of provider confidence, and patient worries about side effects.

Additionally, further statistical analysis of relevant cofactors reveals the influence of demographic and contextual factors, such as age, professional experience, and geographical region, on perception of these barriers. Collectively, the findings emphasize the interplay between systemic, financial, and individual-level challenges, calling for a holistic approach to overcoming obstacles and enhancing IO adoption.

Despite gathering a broad sample and soliciting perspectives from a diverse group of multi-stakeholders on a range of implementation barriers, this study has several limitations. Although the survey instrument was developed through expert consensus [17], formal psychometric validation was not conducted and should be considered in interpreting findings. Sampling bias could not be avoided from our convenience sampling approach and the use of an English-language survey. The modest response rate also raises the possibility of non-response and self-selection bias, as participants with stronger interest or opinions regarding integrative oncology may have been more likely to respond; therefore, findings may not be fully generalizable, including within the MASCC/SIO membership. Selection bias should also be considered, as respondents affiliated with supportive care and integrative oncology societies may have greater awareness and acceptance of integrative oncology, potentially leading to underestimation of implementation barriers in oncology practice. Additionally, healthcare providers in resource-limited settings may not have had access to our survey and may be less likely to participate in professional societies. Future studies may investigate specific barriers and facilitators in different healthcare and resource settings. Moreover, although this survey focused on participants engaged in integrative oncology, future studies should include broader oncology professional populations with less familiarity with integrative oncology to compare perspectives and better characterize implementation barriers across diverse practice settings. We also acknowledge concerns with type I errors as a wide range of analyses was conducted to identify respondent demographic factors associated with the barriers without adjustment for multiple testing.

Despite these limitations, this study is the first to provide preliminary insights about the perspectives of HCP on barriers to the use of IO in cancer supportive care. The findings from this scoping study will be important to shape implementation efforts toward integrating IO into the delivery of cancer care.

## Conclusion

The results of our study provide critical insight into the multifactorial barriers impacting the implementation of integrative oncology (IO) across international healthcare providers who are members of MASCC and/or SIO. High levels of agreement among international stakeholders regarding knowledge, financial, logistical, and individual-level barriers underscore the persistent challenges facing IO integration into standard cancer care. Moreover, the influence of demographic and professional factors such as age, region, and years of experience suggests that perceived barriers are not uniform, but rather contextually shaped. Strategic, system-level solutions that address these interconnected challenges will be essential to supporting the growing interest and clinical utility of IO modalities in oncology care.

## Supplementary Information

Below is the link to the electronic supplementary material.

ESM 1 Supplementary Material 1 (PDF 94.4 KB)

## Data Availability

The datasets used and/or analyzed during the current study are available from the corresponding author on reasonable request.
